# Supplementary material for: Dynamic signature of activity-stability tradeoff in lactamase evolution
Source: Nat Commun. 2026 Jan 21;17:1884. doi: 10.1038/s41467-026-68620-z (PMC12923907; doi:10.1038/s41467-026-68620-z)
Supplement: Supplementary file 1 — Supplementary-Information [file 41467_2026_68620_MOESM1_ESM.pdf]

Dynamic signature of activity-stability tradeoff in lactamase evolution

Ernesto Arcia[1,5], Dimitra Keramisanou[1,5], Lian Jacobs[2], McKenna Parker[1], Julián Meléndez Delgado[3], Vasantha Kumar[1], Sameer Varma[3], Rinat Abzalimov[4], Yu Chen[2]* and Ioannis Gelis[1]*

[1] Department of Chemistry, University of South Florida, Tampa, FL 33620, USA.

[2] Department of Molecular Medicine, Morsani College of Medicine, University of South Florida, Tampa, Florida 33612, United States.

[3] Department of Department of Molecular Biosciences, University of South Florida, Tampa, FL 33620, USA.

[4] Structural Biology Initiative, CUNY Advanced Science Research Center, New York, NY 10031, USA.

[5] These authors contributed equally.

*correspondence to: Ioannis Gelis (igelis@usf.edu) and Yu Chen (ychen1@usf.edu)

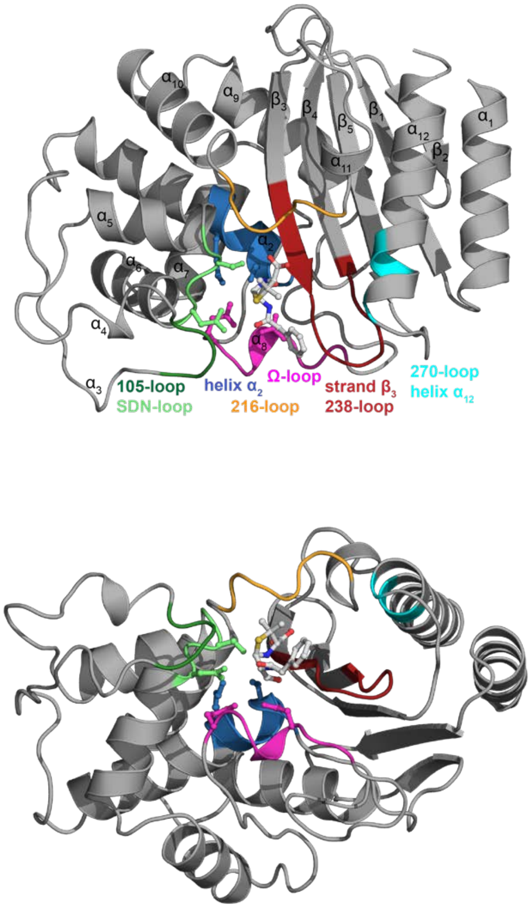

Supplementary Fig. 1: Active site walls of β-lactamases: Overview of the structural elements involved in the catalytic cycle of β-lactamases, highlighting the active site walls in different colors. The core residues S70, K73, S130/N132, E166/N170 that are directly involved in catalysis are shown in sticks, colored as the corresponding walls. Benzylpenicillin, from the acyl enzyme intermediate complex (PBD ID: 1FQG) is shown in white sticks, to indicate the substrate binding site on the structure. Numbering of all secondary structure elements is also marked.

a

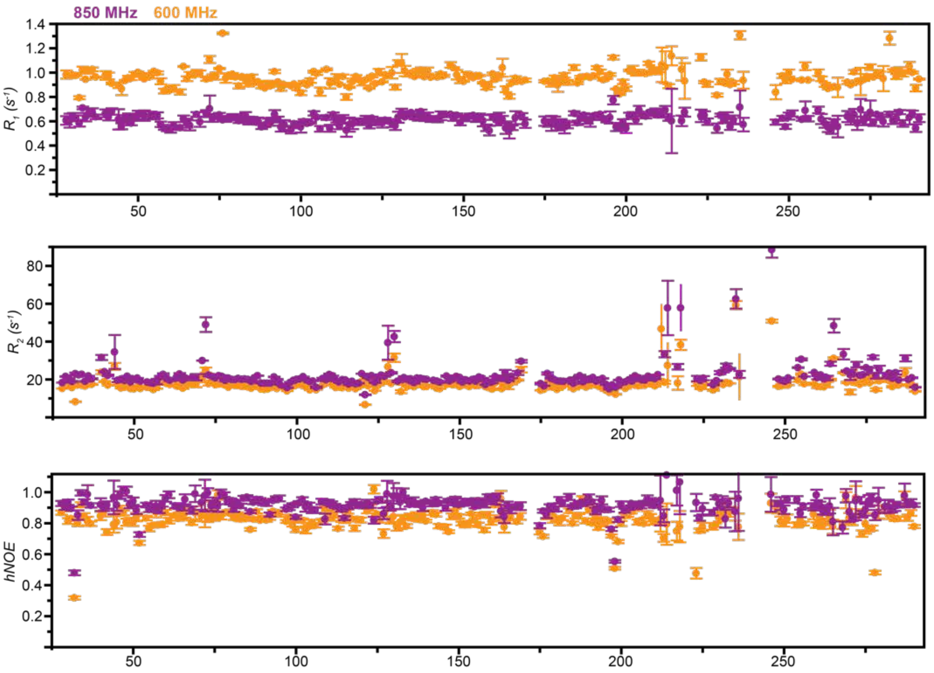

b

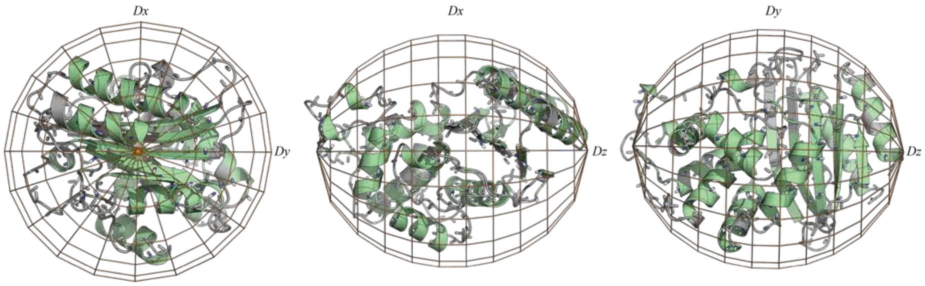

c

Diffusion model	$\lvert AIC_{min}-AIC \rvert$	τ_m (ns)	$D_\parallel/D_\perp$	θ (°)	φ (°)	α (°)	β (°)	γ (°)
Sphere	515.4	12.82	1.00	-	-	-	-	-
Prolate	57.6	12.69	1.17	170.2	185.8	-	-	-
Oblate	157.4	12.71	0.90	63.0	91.0	-	-	-
Ellipsoid	0	12.70	1.16	-	-	99.2	5.3	6.1

local model:

$m0$: {}

$m1$: $\{S^2\}$

$m2$: $\{S^2, \tau_e\}$

$m3$: $\{S^2, R_{ex}\}$

$m4$: $\{S^2, \tau_e, R_{ex}\}$

$m5$: $\{S^2, S_f^2, \tau_s\}$

d

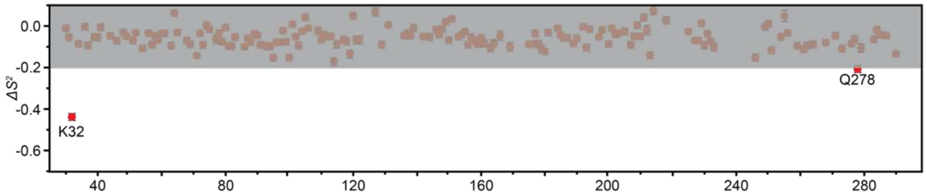

e

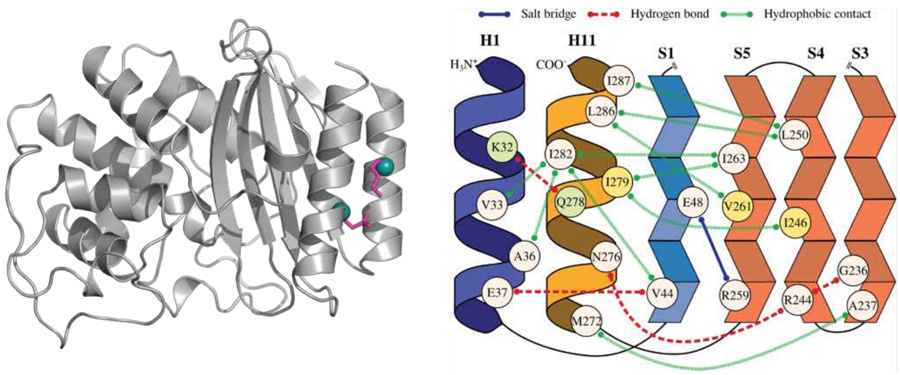

Supplementary Fig. 2: Dynamic properties of G238S **(a)** Relaxation rates R_1 and R_2, and ^{15}N-hNOEs of G238S acquired at two magnetic fields corresponding to 850 and 600 MHz. Errors are propagated from relaxation data measured in duplicate, via Monte Carlo simulations. **(b)** The ellipsoidal diffusion tensor used to minimize the local model-free models $m0$-$m5$. **(c)** Left: the minimized tensor parameters highlighted in yellow. Right: the local models $m0$-$m5$, where S^2 is the squared order parameter, τ is the effective correlation time and R_{ex} is the contribution of slow timescale motions to R_2. **(d)** The difference between the squared order parameter of G238S and TEM-1 ($|\Delta S^2|$) as a function of primary sequence. Most residues show $|\Delta S^2| < 0.1$. Residues with small ΔS^2 values (<0.2) are shaded in gray. TEM-1 S^2 values were obtained from Savard *et al*[1]. Error bars represent error propagation of the difference. **(e)** The two residues, K32 and Q278, showing a large negative ΔS^2 are shown on the structure of TEM-1 with their sidechains depicted in pink is shown on the left. The interaction network connecting the active site with helices α_1 and α_{12} and other regions of the scaffold is shown as a cartoon on the right.

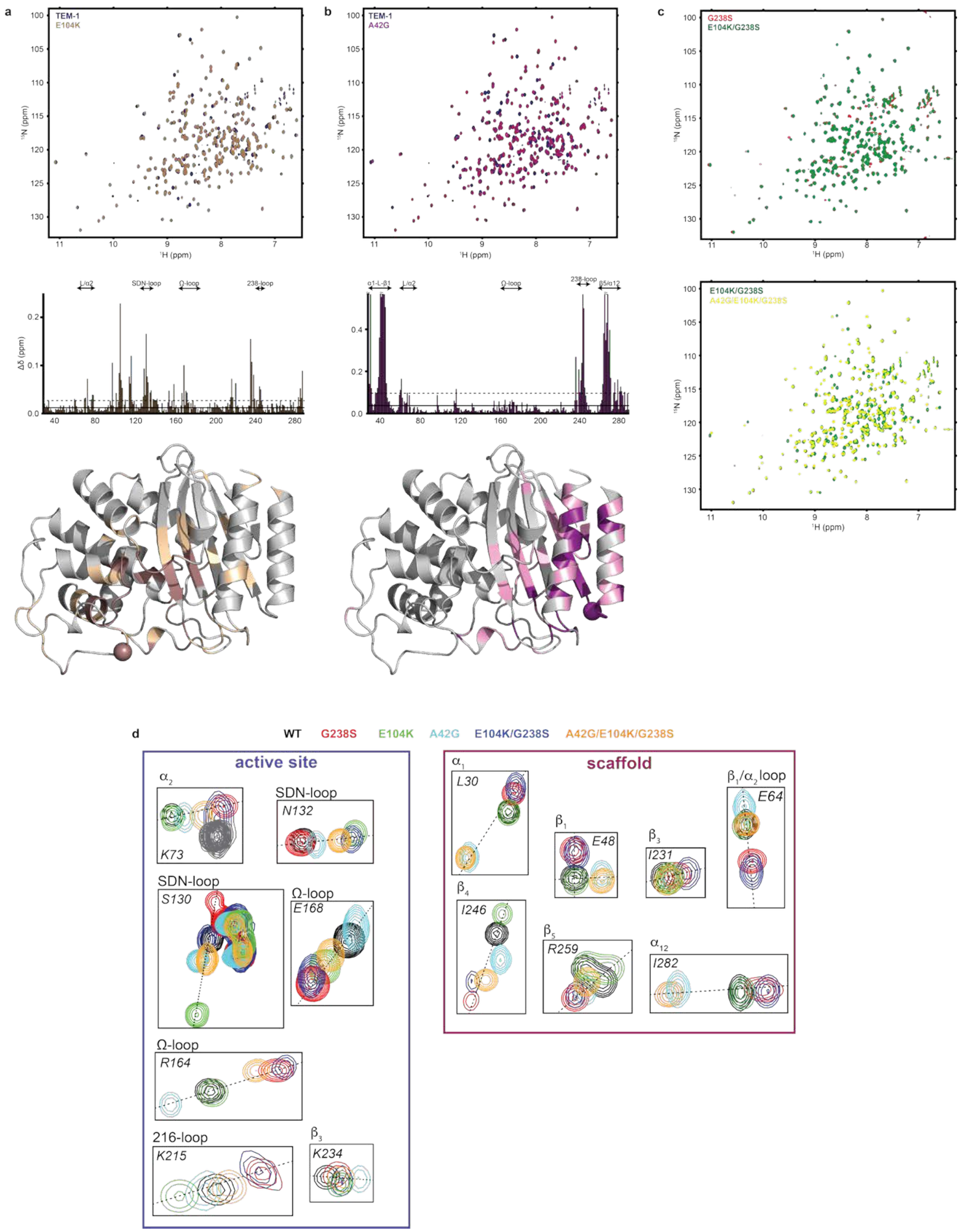

Supplementary Fig. 3: Analysis of TEM-1 evolutionary intermediates by NMR. (a, b) Overlay of the ^{1}H-^{15}N HSQCs of E104 (left) and A42G (right) on the spectrum of TEM-1, together with the corresponding chemical shift perturbation analysis and the mapping on the structure of TEM-1. The site of each mutation is shown as a sphere. **(c)** ^{1}H-^{15}N HSQC overlays of the second (E104K/G238S) onto the first (G238S) (top) and the third (A42G/E104K/G238S) onto the second (bottom) evolutionary intermediate. **(d)** ^{1}H-^{15}N HSQC overlays of all the intermediates along the G238S evolutionary pathway considered in this study, showing a representative set of active site and scaffold residues.

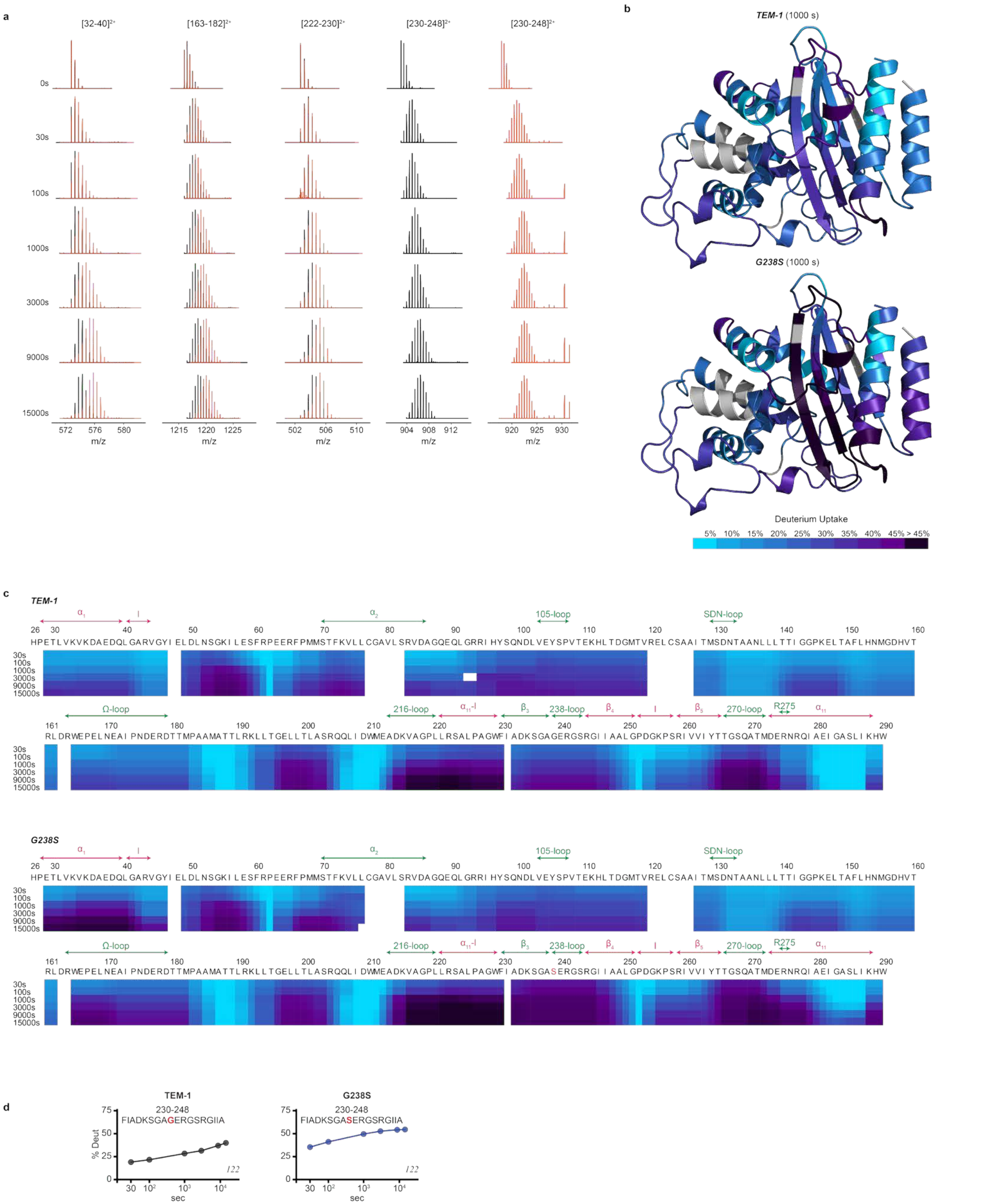

Supplementary Fig. 4: Deuterium incorporation for TEM-1 and G238S. (a) Overlay of TEM-1 (black) and G238S (pink) mass spectra for a representative set of peptides at various labeling time intervals shows that both enzymes exchange via EX2 kinetics. For the last peptide (230-248) the two spectra are shown side by side as

they have a large mass difference due to the mutation in the 238-loop. The spectrum shown at 0 s corresponds to that of the unlabeled peptide. **(b)** Deuterium incorporation for TEM-1 and G238S after labeling for 1000 s mapped on the structure of TEM-1. The color scale is from light blue (<5% uptake) to purple/black (>45% uptake). **(c)** Heatmap showing percent deuterium incorporation for TEM-1 (upper panel) and G238S (lower panel), generated using data acquired at six timepoints from 30 to 15000s. Secondary structure elements comprising active site walls or scaffold sites discussed in the main text are highlighted in green and pink respectively. The color scale is the same used for the heatmap in (b). **(d)** Side-by-side comparison of deuterium uptake for a representative peptide covering the 238-loop in TEM-1 and G238S. Note that the two peptides differ in sequence due to the G238S substitution, which is highlighted in red.

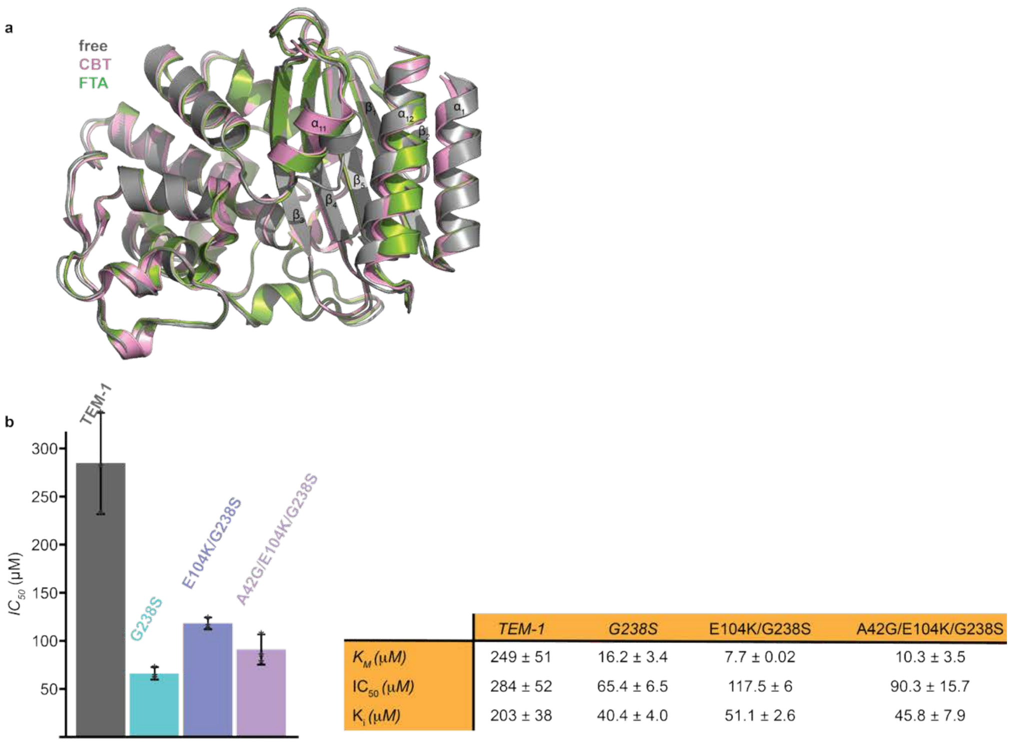

	TEM-1	*G238S*	E104K/G238S	A42G/E104K/G238S
K_M (μM)	249 ± 51	16.2 ± 3.4	7.7 ± 0.02	10.3 ± 3.5
IC_{50} (μM)	284 ± 52	65.4 ± 6.5	117.5 ± 6	90.3 ± 15.7
K_i (μM)	203 ± 38	40.4 ± 4.0	51.1 ± 2.6	45.8 ± 7.9

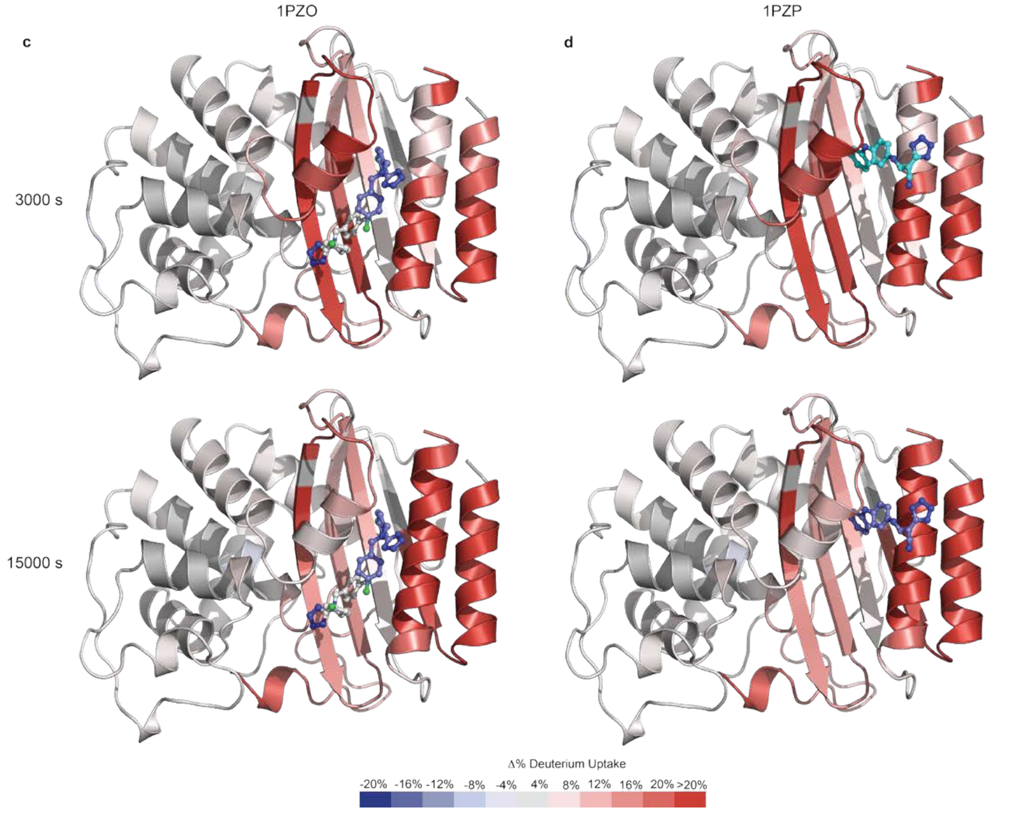

Supplementary Fig. 5: G238S stabilizes the open allosteric site observed in the presence of weak allosteric inhibitors. (a) Structural rearrangements in the presence of allosteric ligands are primarily associated with helix α_{11} and α_{12}. **(b)** Inhibition constants for nitrocefin hydrolysis by the four evolutionary intermediates considered in this study, in the presence of FTA. Dose-response curves containing 10-points were measured in triplicate. Values represent the mean and error bars represent ± one standard deviation of technical replicates.

(c, d) G238S-induced stability changes mapped on structures of wild-type TEM-1 in complex with the allosteric inhibitors CBT (N,N-bis(4-chlorobenzyl)-1H-1,2,3,4-tetraazol-5-amine) (PDB 1PZO) (c) and FTA (3-(4-phenylamino-phenylamino)-2-(1H-tetrazol-5-yl)-acrylonitrile) (PDB 1PZP) (d). The ligands are shown in stick-ball representation. Differential deuterium incorporation is colored with the same scale as in main text Fig. 4.

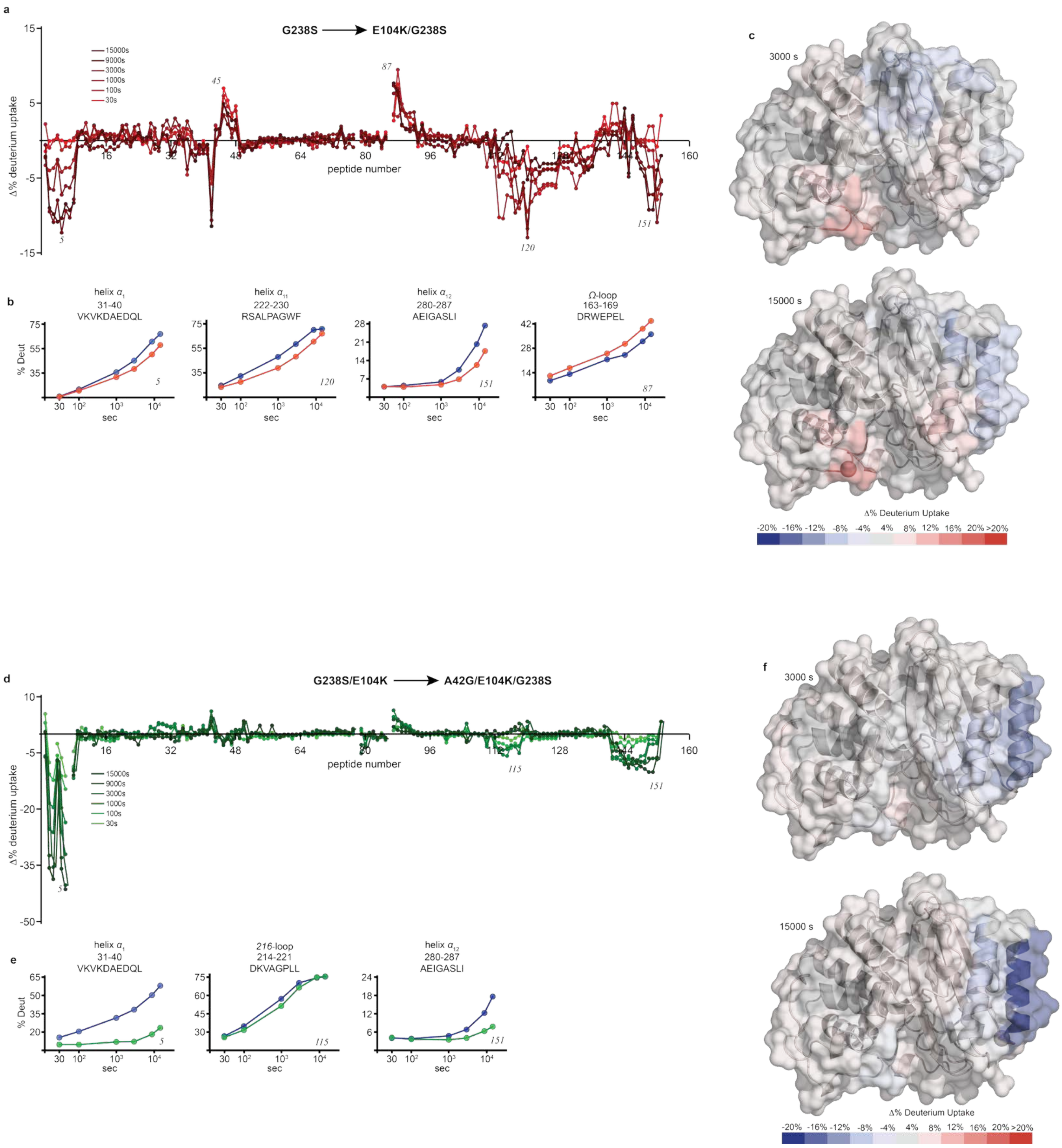

Supplementary Fig. 6: Optimization of local stabilities by secondary substitutions. (a,d) Residual plots expressed as Δ%D between the double mutant E104K/G238S and the initial substitution, G238S ($\%D_{A42G/E104K/G238S}$ - $\%D_{G238S}$), and the triple mutant A42G/E104K/G238S and double mutant E104K/G238S at different timepoints of exchange. Short and long exchange times are shown in light and dark colour, respectively. **(b,e)** Representative examples of deuterium uptake plots as a function of labeling time for the second and third steps of the evolutionary pathway. E104K/G238S and G238S are shown in red and blue, respectively, in (b), and

A42G/E104K/G238S and E104K/G238S are shown in green and blue, respectively in (e). **(c,f)** Differences in deuterium incorporation (Δ%D) at 3000s (top) and 15000s (bottom) plotted on the structure of TEM-1, highlighting how secondary substitutions progressively restore scaffold stability while maintaining the active site dynamics induced by G238S.

References

1 Savard, P. Y. & Gagne, S. M. Backbone dynamics of TEM-1 determined by NMR: evidence for a highly ordered protein. *Biochemistry* **45**, 11414-11424, doi:10.1021/bi060414q (2006).

Table 1: Available MICs, kinetic parameters & stability properties of the mutants included in this study.

Construct	MIC vs CTX	K_{cat} (Ampicillin) (s^{-1})	K_M (Ampicillin) (µM)	Activity (Ampicillin) K_{cat}/K_m ($s^{-1}M^{-1}$)	K_{cat} (Cefotaxime)(s^{-1})	K_M (Cefotaxime)	Activity (Cefotaxime) K_{cat}/K_m ($s^{-1}M^{-1}$)	Stability
WT	5.7 (*)(2)	1210 ± 70 (1)	29.0 ± 5.5 (1)	4.18 x 10^{7} (1)	0.636 ± 0.090 (1)	308 ± 77 (&)(1)	2.07 x 10^{3} (1)	-7.5 ± 0.483 (+) (2)
	0.02 (*)(5)	1300 ± 50 (4)	35 ± 4 (4)	(37 ± 5) x10^{6} (4)	1.7 x 10^{-1} (2)	7.5 x 10^{-4} (!)(2)	(1.5 ± 0.170) x 10^{2} (2)	49.9 ± 0.3 (X) (3)
	0.088 (*)(6)				1 ± 0.5 (3)	1500 ± 900 (&)(3)	(0.68 ± 0.53) x 10^{3} (3)	
	<0.035 (&)(4)				0.25 (7)	450 (&)(7)	(2.0 x 10-3 ± 0.5)x 10^{2} (4)	
					9.0 (8)	6000 (&)(8)	5.6 x 10^{2} (7)	
					2 (9)	1100 (&)(9)	1.5 x 10^{3} (8)(10)	
					2.5 (10)	1684 (&)(10)	1.8 x 10^{3} (9)	
					0.18 (11)	230 (&)(11)	7.8 x 10^{2} (11)	
							3.9 x 10^{3} (12)	
							2.8 x 10^{3} (13)	
							2.1 x 10^{3} (14)	
							1.0 x 10^{3} (15)	
					**0.91 ± 0.04 (17)**	**976 ± 1.5 (17)**	**0.93 x 10^{3} (17)**	
G238S	2.6 x 10^{2} (*)(2)	27.8 ± 0.7 (1)	4.15 ±0.51 (1)	6.70 x 10^{6} (1)	41.8 ± 2.0 (1)	234 ± 25 (&)(1)	1.78 x 10^{5} (1)	-1.94 ± 0.12 (-)(1)
	1.4 (*)(6)	66 ± 1 (4)	4.3 ± 0.4 (4)	(16 ± 2) x10^{6} (4)	(1.43 ± 0.00498) x 10^{1} (2)	(7 ± 1.51) x 10^{-4} (!)(2)	(2.2 ± 0.221) x 10^{4} (2)	-5.3 ± 0.42 (+)(2)
	1.13 (&)(4)				30 ± 8 (3)	240 ± 80 (&)(3)	(130 ± 100) x 10^{3} (3)	43.8 ± 0.1 (X)(3)
					50 ± 3 (4)	190 ± 20 (&)(4)	(2.6 ± 0.03) x 10^{5} (4)	
					66 (8)	290 (&)(8)	2.3 x 10^{5} (8)	
					20 (10)	188 (&)(10)	1.1 x 10^{5} (10)	
					16 (13)	124 (&)(13)	1.3 x 10^{4} (16)	
					50 (15)	403 (&) (15)	1.4 x 10^{6} (13)	
					7.5 (16)	577 (&)(16)	1.3 x 10^{5} (15)	
					**15.2 ± 6.8 (17)**	**490 ± 6 (17)**	**3 x 10^{4} (17)**	
E104K	11 (*)(2)	499 ± 36 (1)	19.5 ± 3.7 (1)	2.56 x 10^{7} (1)	9.25 ± 1.9 (1)	980 ± 256 (&)(1)	9.44 x 10^{3}(1)	-0.22 ± 0.04 (-)(1)

	0.08 (*)(5)	1200 ± 60 (4)	39 ± 6 (4)	(30 ± 5) x10^{6} (4)	3.9 ± 0.18 (2)	(5 ± 2.9) x 10^{-3} (!)(2)	(6 ± 1.05) x 10^{2} (2)	-7.1 ± 0.62 (+)(2)
	0.13 (*)(6)				1.0 ± 0.6 (3)	750 ± 560 (&)(3)	(1.3 ± 1.2) x 10^{3} (3)	49.4 ± 0.1 (X)(3)
	0.07 (&)(4)				2.5 (7)	470 (&)(7)	(1.2 x 10^{-2} ± 0.4) x 10^{3} (4)	
					25 (9)	1000 (&)(9)	5.3 x 10^{3} (7)	
							2.5 x 10^{4} (9)	
A42G	5.7 (*)(2)						(2.0 ± 0.498) x 10^{2} (2)	-11 ± 0.68 (+)(2)
	0.088 (*)(6)							
G238S/E104K	2.0 x 10^{3} (*)(2)	8.08 ± 0.72 (1)	5.11 ±2.33 (1)	1.58 x 10^{6} (1)	29.9 ± 0.8 (1)	17.9 ± 5.4 (&)(1)	1.67 x 10^{6} (1)	-2.24 ± 0.13 (-)(1)
	10 (*)(5)	38 ± 2 (4)	2.3 ± 0.5 (4)	(17 ± 4) x10^{6} (4)	(9.21 ± 7.43)x 10^{-3} (2)	(4.6 ± 0.376) x10^{-4} (!)(2)	(2.0 ± 0.133) x 10^{4} (2)	-4.4 ± 0.29 (+)(2)
	18 (&)(4)				33 ± 9 (3)	150 ± 50 (&)(3)	(220 ± 90) x 10^{3} (3)	43.5 ± 0.1 (X)(3)
					87 ± 4 (4)	31 ± 5 (&) (4)	(2.8 ± 0.4) x 10^{6} (4)	
					**27.6 ± 2.3 (17)**	**210 ± 25 (17)**	**13.1 x 10^{4} (17)**	
G238S/E104K/A42G	2.9 x 10^{2} (*)(2)				(2.61 ± 0.004)x10^{1} (2)	(1.1 ± 0.241)x10^{-4} (!)(2)	(2.2 ± 0.222) x 10^{5} (2)	-5.3 ± 0.27 (+)(2)
					**43 ± 2 (17)**	**188 ± 3 (17)**	**23.0 x 10^{4} (17)**	

Symbols: (+) ΔG (kcal/mol); (-) ΔΔG (kcal/mol); (X) Tm (∘C); (*) μg/mL; (!) M; (&) μM

[1]ΔΔG given as the difference from the WT

[2]25 C, MIC broth microdilution method, ΔΔG of unfolding was calculated from the van't Hoff enthalpy at 25 °C as ΔG = ΔH(1 – T/Tm), *kinetic parameters normalized by μg total soluble protein*

[3]Kcat/KM reported as mM^{-1}s^{-1} data in original source, converted to M^{-1}s^{-1} for clarity

[4]Data obtained using DH5α cells. Data for K_{cat}/K_M Ampicillin refers to Benzylpenicillin. K_{cat}/K_M reported as μM^{-1}s^{-1} in original source, converted to M^{-1}s^{-1} for clarity.

[5]MIC (24h, 37 C)

[6]Median of three replicates. 20h, 35 C

[7]Buffer: 0.1 M phosphate, pH 7, 25 C

[8]Buffer: 50 mM phosphate, pH 7, 30 C

[9]Buffer: pH 7.0, 30 C

[10]Buffer: 10 mM sodium bicarbonate, pH 7.0, 37 C

[11]Buffer: 50 mM phosphate, 100 mM NaCl, pH 7.0, 37 C

[12]Buffer: 50 mM phosphate, pH 7.0, 30 C

[13]Buffer: 50 mM phosphate, pH 7.0, 30 C

[14]Buffer: 50 mM phosphate, pH 7.0, 30 C

[15]Buffer: 100 mM phosphate, pH 7.0, 25 C

[16]Buffer: 50 mM phosphate, pH 7.0, 30 C

[17]This study

(1) Wang, X., Minasov, G. & Shoichet, B. K. Evolution of an antibiotic resistance enzyme constrained by stability and activity trade-offs. J Mol Biol 320, 85-95, doi:10.1016/S0022-2836(02)00400-X (2002).

(2) Knies, J. L., Cai, F. & Weinreich, D. M. Enzyme Efficiency but Not Thermostability Drives Cefotaxime Resistance Evolution in TEM-1 beta-Lactamase. Mol Biol Evol 34, 1040-1054, doi:10.1093/molbev/msx053 (2017).

(3) Alejaldre, L. et al. Known Evolutionary Paths Are Accessible to Engineered ss-LactamasesHaving Altered Protein Motions at the Timescale of Catalytic Turnover. Front Mol Biosci7, 599298, doi:10.3389/fmolb.2020.599298 (2020).

(4) Hart, K. M., Ho, C. M., Dutta, S., Gross, M. L. & Bowman, G. R. Modelling proteins' hidden conformations to predict antibiotic resistance. Nat Commun 7, 12965,doi:10.1038/ncomms12965 (2016).

(5) Stemmer, W. P. Rapid evolution of a protein in vitro by DNA shuffling. Nature 370, 389-391, doi:10.1038/370389a0 (1994).

(6) Weinreich, D. M., Delaney, N. F., Depristo, M. A. & Hartl, D. L. Darwinian evolution can follow only very few mutational paths to fitter proteins. Science 312, 111-114, doi:10.1126/science.1123539 (2006).

(7) Sowek, J. A., Singer, S. B., Ohringer, S., Malley, M. F., Dougherty, T. J., Gougoutas, J. Z., & Bush, K. (1991). Substitution of lysine at position 104 or 240 of TEM-1PTZ18R .beta.-lactamase enhances the effect of serine-164 substitution on hydrolysis or affinity for cephalosporins and the MONOBACTAM aztreonam. Biochemistry, 30(13), 3179–3188. https://doi.org/10.1021/bi00227a004

(8) Raquet, X., Lamotte-Brasseur, J., Fonze, E., Goussard, S., Courvalin, P., and Frere, J. M. (1994). TEM beta-lactamase mutants hydrolysing third-generation cephalosporins-A kinetic and molecular modelling analysis. J. Mol. Biol. 244, 625–639. doi: 10.1006/jmbi.1994.1756

(9) Petit, A., Maveyraud, L., Lenfant, F., Samama, J. P., Labia, R., and Masson, J. M. (1995). Multiple substitutions at position 104 of ß-lactamase TEM-1: assessing the role of this residue in substrate specificity. Biochem. J. 305, 33–40. doi: 10.1042/bj3050033

(10) Saves, I. et al. Mass spectral kinetic study of acylation and deacylation during the hydrolysis of penicillins and cefotaxime by beta-lactamase TEM-1 and the G238S mutant.Biochemistry 34, 11660-11667, doi:10.1021/bi00037a003 (1995).

(11) Vakulenko, S. B. et al. Effects on substrate profile by mutational substitutions at positions 164 and 179 of the class A TEM(pUC19) beta-lactamase from Escherichia coli. J Biol Chem 274, 23052-23060, doi:10.1074/jbc.274.33.23052 (1999).

(12) Venkatachalam, K. V., Huang, W., Larocco, M., and Palzkill, T. (1994). Characterization of TEM-1 ß-lactamase mutants from positions 238 to 241 with increased catalytic efficiency for ceftazidime. J. Biol. Chem. 269, 23444–23450.

(13) Cantu, C., & Palzkill, T. (1998). The role of residue 238 of TEM-1 β-lactamase in the hydrolysis of extended-spectrum antibiotics. Journal of Biological Chemistry, 273(41), 26603–26609. https://doi.org/10.1074/jbc.273.41.

(14) Brown, N. G., Pennington, J. M., Huang, W., Ayvaz, T., and Palzkill, T. (2010). Multiple global suppressors of protein stability defects facilitate the evolution of extended-spectrum TEM β-lactamases. J. Mol. Biol. 404, 832–846. doi: 10.1016/j.jmb.2010.10.008

(15) Dellus-Gur, E., Elias, M., Caselli, E., Prati, F., Salverda, M. L. M., De Visser, J. A. G. M., et al. (2015). Negative epistasis and evolvability in TEM-1 β-lactamase - the thin line between an enzyme's conformational freedom and disorder. J. Mol. Biol. 427, 2396–2409. doi: 10.1016/j.jmb.2015.05.011

(16) Viadiu, H., Osuna, J., Fink, A. L., and Soberon, X. (1995). A new TEM β-lactamase double mutant with broadened specificity reveals substrate-dependent functional interactions. J. Biol. Chem. 270, 781–787. doi: 10.1074/jbc.270.2.781

Table 2: Relaxation analysis of the TEM G238S mutant. R1 (s^{-1}), R2 (s^{-1}) and $\{^1H\}-^{15}N$ NOE are presented for two fields. The model free analysis is performed as describe in the methods and the best fit model for each residue is shown with its respective second order parameters. CPMG values are also presented with the best fit model and the values of $R^2{}_{eff}$ (Hz) and k_{ex} (s^{-1}) and their respective errors. Residues marked with an (a) are unassigned, (b) tentatively assigned, (c) overlap and (d/e) broad/poor fitting.

	**600 MHz**			**850 MHz**			**Model Free**				**CPMG**				
Residue	R1	R2	NOE	R1	R2	NOE	Model	S^2	S^2_f	S^2_s	$R^2{}_{eff}$	$\delta R^2{}_{eff}$	k_{ex}	δk_{ex}	Model
HIS26a	-	-	-	-	-	-	-	-	-	-	-	-	-	-	-
PRO27	-	-	-	-	-	-	-	-	-	-	-	-	-	-	-
GLU28	0.98	15.26	0.87	0.61	18.33	0.93	m1	0.79	-	-	-	-	-	-	-
THR29	0.97	18.04	0.89	0.61	21.56	0.91	m3	0.85	-	-	4.38	0.35	1860.56	257.19	LM63
LEU30	0.98	17.49	0.81	0.64	20.61	0.94	m1	0.88	-	-	-	-	-	-	-
VAL31	0.98	15.71	0.83	0.62	18.93	0.89	m1	0.85	-	-	-	-	-	-	-
LYS32	0.79	8.25	0.32	0.58	23.07	0.48	m5	0.47	0.62	0.77	-	-	-	-	-
VAL33	1.02	17.38	0.80	0.71	22.34	0.85	-	-	-	-	-	-	-	-	-
LYS34	0.94	16.93	0.92	0.63	22.74	1.00	m3	0.84	-	-	4.56	0.33	970.25	99.43	LM63
ASP35	1.01	16.50	0.85	0.67	19.04	0.94	m1	0.83	-	-	-	-	-	-	-
ALA36	1.03	18.38	0.81	0.68	21.85	0.99	m3	0.90	-	-	-	-	-	-	-
GLU37	0.96	17.44	0.80	0.64	20.34	0.91	m3	0.85	-	-	-	-	-	-	-
ASP38c	1.33	23.73	0.77	1.13	39.29	0.82	-	-	-	-	-	-	-	-	-
GLN39c	1.10	17.25	0.93	0.68	19.34	0.88	-	-	-	-	-	-	-	-	-
LEU40	0.98	23.85	0.85	0.66	31.61	0.92	m3	0.87	-	-	4.01	1.25	911.82	751.74	LM63
GLY41	0.97	18.91	0.86	0.64	24.07	0.90	m3	0.90	-	-	10.22	0.81	775.15	77.43	LM63
ALA42	0.91	17.02	0.74	0.68	22.18	0.92	m4	0.82	-	-	-	-	-	-	-
ARG43b	2.16	30.59	0.35	2.59	35.52	0.56	-	-	-	-	-	-	-	-	-
VAL44	0.89	26.87	0.79	0.62	34.52	0.97	m3	0.82	-	-	14.19	8.05	2736.41	3274.93	LM63
GLY45	0.87	17.69	0.81	0.56	19.57	0.92	m1	0.83	-	-	-	-	-	-	-
TYR46	0.93	15.49	0.89	0.62	20.17	0.98	m3	0.82	-	-	-	-	-	-	-
ILE47	0.96	15.81	0.87	0.63	19.48	1.02	m1	0.84	-	-	-	-	-	-	-
GLU48	0.95	17.77	0.81	0.58	21.70	1.01	m3	0.85	-	-	8.13	0.58	965.79	125.12	LM63
LEU49	0.98	15.15	0.82	0.63	18.61	0.92	m1	0.80	-	-	-	-	-	-	-
ASP50	1.00	15.04	0.80	0.65	20.00	0.94	m5	0.80	0.84	0.96	-	-	-	-	-
LEU51	0.99	16.15	0.84	0.66	20.74	0.88	m3	0.86	-	-	-	-	-	-	-
ASN52	0.95	15.20	0.67	0.65	19.04	0.73	m5	0.80	0.84	0.95	-	-	-	-	-
SER53c	0.93	15.82	0.78	0.61	19.06	0.88	-	-	-	-	-	-	-	-	-
GLY54	0.97	15.83	0.83	0.65	18.65	0.92	m1	0.83	-	-	-	-	-	-	-
LYS55	1.01	15.71	0.77	0.64	18.26	0.89	m5	0.78	0.82	0.95	-	-	-	-	-
ILE56	1.00	14.66	0.78	0.65	17.99	0.91	m5	0.79	0.82	0.96	-	-	-	-	-

Continued on next page

Table 2 – continued from previous page

	600 MHz			850 MHz			Model Free				CPMG				
Residue	R1	R2	NOE	R1	R2	NOE	Model	S^2	S^2_f	S^2_s	R^2_{eff}	$\delta R^2_{\mathrm{eff}}$	k_{ex}	δk_{ex}	Model
LEU57	0.93	16.16	0.87	0.57	18.85	0.91	m1	0.83	-	-	-	-	-	-	-
GLU58	0.85	18.61	0.79	0.54	21.35	0.88	m3	0.81	-	-	-	-	-	-	-
SER59	0.87	18.60	0.79	0.54	22.71	0.93	m3	0.80	-	-	-	-	-	-	-
PHE60	0.86	17.89	0.87	0.53	20.42	0.96	m3	0.80	-	-	-	-	-	-	-
ARG61	0.87	17.35	0.82	0.56	19.53	0.98	m1	0.83	-	-	-	-	-	-	-
PRO62	-	-	-	-	-	-	-	-	-	-	-	-	-	-	-
GLU63	0.84	16.63	0.85	0.55	20.84	0.91	m3	0.78	-	-	-	-	-	-	-
GLU64	1.05	18.54	0.83	0.62	22.88	0.91	m3	0.93	-	-	4.20	0.32	2291.39	294.27	LM63
ARG65	0.95	15.42	0.85	0.58	19.65	0.90	m1	0.84	-	-	-	-	-	-	-
PHE66	0.98	17.58	0.87	0.57	20.69	0.95	m3	0.87	-	-	-	-	-	-	-
PRO67	-	-	-	-	-	-	-	-	-	-	-	-	-	-	-
MET68	-	-	-	0.66	21.30	0.88	m1	0.91	-	-	-	-	-	-	-
MET69	0.99	17.45	0.87	0.59	21.19	0.99	m1	0.87	-	-	-	-	-	-	-
SER70b	-	-	-	-	-	-	-	-	-	-	-	-	-	-	-
THR71	0.95	20.30	0.85	0.66	29.97	0.93	m3	0.85	-	-	11.45	1.81	2141.43	401.38	LM63
PHE72	1.11	24.96	0.86	0.70	48.97	0.98	m3	0.95	-	-	6.05	3.34	2266.34	2951.70	LM63
LYS73	0.97	17.77	0.83	0.63	22.30	1.00	m3	0.85	-	-	-	-	-	-	-
VAL74	0.97	19.02	0.86	0.64	21.61	0.91	m1	0.92	-	-	-	-	-	-	-
LEU75	1.03	17.19	0.83	0.65	20.81	0.92	m1	0.91	-	-	-	-	-	-	-
LEU76	1.32	17.55	0.98	0.61	22.02	0.92	m5	0.92	1.00	0.92	5.03	1.08	2076.56	557.99	LM63
CYS77	0.95	17.63	0.84	0.62	21.64	0.99	m3	0.88	-	-	-	-	-	-	-
GLY78	0.98	17.10	0.87	0.63	19.48	0.98	m1	0.89	-	-	-	-	-	-	-
ALA79	0.99	15.85	0.87	0.63	20.69	0.94	m1	0.86	-	-	-	-	-	-	-
VAL80	0.96	18.05	0.87	0.63	20.07	0.94	m3	0.90	-	-	-	-	-	-	-
LEU81	0.89	16.84	0.83	0.60	19.92	0.89	m1	0.84	-	-	-	-	-	-	-
SER82	0.97	16.70	0.86	0.62	20.52	0.93	m3	0.85	-	-	-	-	-	-	-
ARG83	0.96	16.36	0.83	0.65	20.04	0.91	m3	0.86	-	-	-	-	-	-	-
VAL84	0.91	16.34	0.84	0.61	20.29	0.96	m3	0.82	-	-	-	-	-	-	-
ASP85	0.97	16.02	0.83	0.61	20.01	0.97	m1	0.85	-	-	-	-	-	-	-
ALA86	0.92	15.37	0.76	0.61	18.94	0.87	m4	0.79	-	-	-	-	-	-	-
GLY87	0.92	16.44	0.83	0.60	20.86	0.94	m3	0.82	-	-	-	-	-	-	-
GLN88	0.89	17.78	0.88	0.55	22.00	0.95	m3	0.82	-	-	-	-	-	-	-
GLU89	0.87	14.94	0.82	0.57	18.07	0.95	m1	0.79	-	-	-	-	-	-	-
GLN90	0.91	14.74	0.83	0.59	18.04	0.93	m1	0.79	-	-	-	-	-	-	-
LEU91	-	-	-	0.60	18.01	0.93	m1	0.79	-	-	-	-	-	-	-

Continued on next page

Table 2 – continued from previous page

	600 MHz			850 MHz			Model Free				CPMG				
Residue	R1	R2	NOE	R1	R2	NOE	Model	S^2	S^2_f	S^2_s	R^2_{eff}	δR^2_{eff}	k_{ex}	δk_{ex}	Model
GLY92	1.01	15.66	0.83	0.66	18.02	0.86	m5	0.78	0.82	0.95	-	-	-	-	-
ARG93	0.92	16.89	0.88	0.62	21.34	0.95	m3	0.83	-	-	-	-	-	-	-
ARG94	0.87	15.19	0.87	0.55	19.95	0.96	m3	0.78	-	-	-	-	-	-	-
ILE95	0.90	16.55	0.86	0.57	17.24	0.96	m1	0.75	-	-	-	-	-	-	-
HIS96c	0.47	13.18	6.32	-	-	-	-	-	-	-	-	-	-	-	-
TYR97	0.89	13.93	0.81	0.53	15.67	0.89	m5	0.70	0.75	0.93	-	-	-	-	-
SER98	0.90	15.55	0.84	0.58	18.37	0.90	m1	0.80	-	-	-	-	-	-	-
GLN99	0.88	16.61	0.79	0.57	19.53	0.92	m1	0.80	-	-	-	-	-	-	-
ASN100	0.83	17.30	0.76	0.55	20.10	0.84	m4	0.77	-	-	-	-	-	-	-
ASP101	0.91	17.34	0.82	0.60	20.28	0.93	m1	0.92	-	-	-	-	-	-	-
LEU102	0.90	17.17	0.82	0.58	21.46	0.95	m3	0.83	-	-	-	-	-	-	-
VAL103	0.93	15.86	0.76	0.59	19.72	0.90	m3	0.81	-	-	-	-	-	-	-
GLU104	-	-	-	0.67	18.89	0.92	m1	0.83	-	-	-	-	-	-	-
TYR105	-	-	-	0.63	19.20	0.88	m1	0.84	-	-	5.18	0.39	1947.99	182.66	LM63
SER106	0.84	14.56	0.79	0.54	15.87	0.88	m1	0.80	-	-	-	-	-	-	-
PRO107	-	-	-	-	-	-	-	-	-	-	-	-	-	-	-
VAL108	1.03	16.97	0.81	0.63	20.51	0.94	m1	0.91	-	-	-	-	-	-	-
THR109	0.91	17.80	0.88	0.54	19.92	0.83	m3	0.84	-	-	-	-	-	-	-
GLU110	0.93	18.87	0.86	0.65	22.64	0.93	m3	0.89	-	-	-	-	-	-	-
LYS111	0.95	17.73	0.83	0.61	21.96	0.92	m3	0.86	-	-	-	-	-	-	-
HIS112	0.90	15.72	0.86	0.56	19.65	0.91	m3	0.81	-	-	-	-	-	-	-
LEU113	0.97	15.90	0.77	0.62	18.64	0.90	m5	0.85	0.86	0.99	-	-	-	-	-
THR114	0.80	15.59	0.82	0.53	19.89	0.89	m3	0.72	-	-	-	-	-	-	-
ASP115	0.88	16.97	0.82	0.57	19.76	0.83	m4	0.79	-	-	-	-	-	-	-
GLY116	0.93	15.40	0.87	0.58	17.61	0.90	m1	0.80	-	-	-	-	-	-	-
MET117	0.97	16.60	0.83	0.60	19.01	0.93	m1	0.83	-	-	-	-	-	-	-
THR118	0.94	15.11	0.77	0.60	17.72	0.94	m1	0.82	-	-	-	-	-	-	-
VAL119	0.92	16.04	0.83	0.60	18.01	0.92	m1	0.78	-	-	-	-	-	-	-
ARG120	0.94	19.01	0.82	0.61	23.06	0.92	m1	0.98	-	-	-	-	-	-	-
GLU121d	0.87	6.72	-	0.57	11.93	0.91	-	-	-	-	-	-	-	-	-
LEU122	0.97	16.62	0.88	0.62	21.04	0.93	m3	0.86	-	-	-	-	-	-	-
CYS123	0.91	18.14	0.90	0.59	22.00	0.92	m3	0.81	-	-	-	-	-	-	-
SER124	1.01	14.06	1.02	0.59	18.48	0.82	m1	0.74	-	-	-	-	-	-	-
ALA125	0.92	16.89	0.86	0.59	20.87	0.94	-	-	-	-	7.52	0.60	2590.56	207.14	LM63
ALA126	0.98	18.63	0.82	0.61	22.25	0.95	m3	0.87	-	-	4.65	0.35	2317.52	250.31	LM63

Continued on next page

Table 2 – continued from previous page

	**600 MHz**			**850 MHz**			**Model Free**				**CPMG**				
Residue	R1	R2	NOE	R1	R2	NOE	Model	S^2	S^2_f	S^2_s	$R^2{}_{eff}$	$\delta R^2{}_{eff}$	k_{ex}	δk_{ex}	Model
ILE127	0.97	19.39	0.73	0.60	21.64	0.86	m1	0.94	-	-	-	-	-	-	-
THR128	1.00	26.69	0.89	0.58	39.43	0.99	m3	0.88	-	-	17.90	6.84	1302.68	540.18	LM63
MET129	0.88	17.84	0.82	0.56	23.53	0.91	m3	0.79	-	-	6.39	0.67	1408.37	391.78	CR72 full
SER130	1.08	31.47	0.84	0.63	42.66	0.98	m3	0.93	-	-	12.65	2.76	-	-	-
ASP131	1.08	18.72	0.78	0.67	19.07	0.98	m1	1.00	-	-	9.10	1.61	625.70	222.64	LM63
ASN132	1.01	13.56	0.81	0.63	19.96	0.94	m1	0.85	-	-	-	-	-	-	-
THR133	-	-	-	0.66	20.93	1.00	m1	0.90	-	-	-	-	-	-	-
ALA134	-	-	-	0.67	19.94	0.95	m1	0.88	-	-	-	-	-	-	-
ALA135	0.97	16.86	0.84	0.63	20.31	0.92	m3	0.84	-	-	-	-	-	-	-
ASN136	1.01	16.16	0.83	0.64	18.28	0.95	m1	0.88	-	-	-	-	-	-	-
LEU137	1.02	17.31	0.85	0.68	20.74	0.93	m1	0.91	-	-	-	-	-	-	-
LEU138	0.97	17.87	0.82	0.63	20.63	0.92	m3	0.85	-	-	-	-	-	-	-
LEU139	1.02	16.17	0.85	0.64	20.22	0.90	m1	0.89	-	-	-	-	-	-	-
THR140	0.96	16.23	0.81	0.62	20.05	0.94	m3	0.83	-	-	-	-	-	-	-
THR141c	1.00	11.95	0.89	0.73	14.29	0.80	-	-	-	-	-	-	-	-	-
ILE142	0.96	16.86	0.87	0.62	19.53	0.92	m1	0.85	-	-	-	-	-	-	-
GLY143	0.99	15.36	0.86	0.64	19.18	0.95	m1	0.84	-	-	-	-	-	-	-
GLY144	0.97	15.07	0.78	0.65	18.58	0.93	m1	0.83	-	-	-	-	-	-	-
PRO145	-	-	-	-	-	-	-	-	-	-	-	-	-	-	-
LYS146	1.06	17.33	0.78	0.66	21.80	0.90	m5	0.90	0.92	0.98	-	-	-	-	-
GLU147	1.04	17.66	0.75	0.62	19.21	0.94	m5	0.84	0.88	0.96	-	-	-	-	-
LEU148	0.98	17.20	0.84	0.65	20.17	0.93	m1	0.92	-	-	-	-	-	-	-
THR149	0.94	17.71	0.88	0.59	21.56	0.95	m1	0.93	-	-	-	-	-	-	-
ALA150	0.93	16.31	0.84	0.62	21.12	0.92	m3	0.84	-	-	-	-	-	-	-
PHE151	0.98	17.02	0.80	0.62	18.59	0.93	m1	0.92	-	-	-	-	-	-	-
LEU152	0.98	17.00	0.79	0.64	19.73	0.94	m3	0.88	-	-	-	-	-	-	-
HIS153	0.96	16.26	0.80	0.65	19.15	0.93	m1	0.85	-	-	-	-	-	-	-
ASN154	0.98	17.15	0.86	0.62	19.97	0.94	m1	0.86	-	-	-	-	-	-	-
MET155	0.97	16.71	0.82	0.64	19.05	0.91	m1	0.84	-	-	-	-	-	-	-
GLY156	0.92	16.87	0.79	0.60	21.26	0.94	m3	0.83	-	-	-	-	-	-	-
ASP157	0.87	17.26	0.86	0.55	20.63	0.93	m3	0.85	-	-	-	-	-	-	-
HIS158	0.90	16.20	0.76	0.52	17.27	0.92	m5	0.71	0.76	0.94	-	-	-	-	-
VAL159	-	-	-	0.63	18.61	0.97	m1	0.81	-	-	-	-	-	-	-
THR160	0.95	14.90	0.81	0.61	18.66	0.96	m1	0.82	-	-	-	-	-	-	-
ARG161	0.98	18.14	0.92	0.63	21.59	0.94	m3	0.86	-	-	-	-	-	-	-

Continued on next page

Table 2 – continued from previous page

	600 MHz			850 MHz			Model Free				CPMG				
Residue	R1	R2	NOE	R1	R2	NOE	Model	S^2	S^2_f	S^2_s	$R^2{}_{\text{eff}}$	$\delta R^2{}_{\text{eff}}$	k_{ex}	δk_{ex}	Model
LEU162	1.04	19.38	0.83	0.61	20.68	0.97	m1	1.00	-	-	-	-	-	-	-
ASP163	0.86	15.16	0.96	0.54	20.61	0.88	m3	0.75	-	-	-	-	-	-	-
ARG164	0.80	17.28	0.78	0.53	23.57	0.84	m4	0.73	-	-	-	-	-	-	-
TRP165	0.92	16.26	0.87	0.55	19.56	0.92	m3	0.80	-	-	-	-	-	-	-
GLU166	0.95	18.17	0.84	0.63	21.68	0.89	m3	0.85	-	-	-	-	-	-	-
PRO167	-	-	-	-	-	-	-	-	-	-	-	-	-	-	-
GLU168	0.93	19.68	0.81	0.65	23.43	0.91	m3	0.86	-	-	5.07	0.44	3042.43	403.75	LM63
LEU169	0.94	25.20	0.84	0.58	29.59	0.91	m3	0.85	-	-	-	-	-	-	-
ASN170b	0.74	11.24	0.77	0.35	15.52	0.75	-	-	-	-	-	-	-	-	-
GLU171b	0.94	19.81	0.81	0.65	20.26	0.94	-	-	-	-	-	-	-	-	-
ALA172a	-	-	-	-	-	-	-	-	-	-	-	-	-	-	-
ILE173b	0.77	10.26	0.79	0.37	18.47	0.84	-	-	-	-	-	-	-	-	-
PRO174	-	-	-	-	-	-	-	-	-	-	-	-	-	-	-
ASN175	0.93	14.27	0.75	0.60	17.84	0.78	m5	0.76	0.80	0.96	-	-	-	-	-
ASP176	0.93	14.78	0.71	0.59	16.67	0.88	m5	0.75	0.78	0.96	-	-	-	-	-
GLU177	0.91	18.14	0.85	0.58	23.17	0.86	m3	0.81	-	-	-	-	-	-	-
ARG178	0.96	16.33	0.85	0.60	20.63	0.91	m3	0.84	-	-	-	-	-	-	-
ASP179	0.90	16.03	0.85	0.56	20.12	0.90	m3	0.83	-	-	-	-	-	-	-
THR180	0.93	14.71	0.83	0.58	19.30	0.89	m3	0.78	-	-	-	-	-	-	-
THR181	0.98	17.12	0.81	0.55	19.10	0.95	m1	0.87	-	-	-	-	-	-	-
MET182	0.92	16.61	0.83	0.57	19.04	0.93	m1	0.84	-	-	-	-	-	-	-
PRO183	-	-	-	-	-	-	-	-	-	-	-	-	-	-	-
ALA184	1.04	17.33	0.88	0.67	19.65	0.97	m1	0.91	-	-	-	-	-	-	-
ALA185	0.91	15.07	0.80	0.59	17.93	0.91	m1	0.79	-	-	-	-	-	-	-
MET186	0.96	16.90	0.85	0.62	20.98	0.91	m3	0.87	-	-	-	-	-	-	-
ALA187	0.96	17.43	0.84	0.65	21.93	0.93	m3	0.88	-	-	-	-	-	-	-
THR188	0.97	16.65	0.96	0.61	18.69	0.92	m1	0.85	-	-	-	-	-	-	-
THR189	0.96	15.93	0.82	0.61	20.02	0.94	m3	0.84	-	-	-	-	-	-	-
LEU190	0.97	17.08	0.89	0.64	20.30	0.97	m1	0.86	-	-	-	-	-	-	-
ARG191	1.04	18.40	0.80	0.63	21.05	0.94	m1	0.91	-	-	-	-	-	-	-
LYS192	1.01	17.00	0.83	0.66	19.06	0.88	m1	0.90	-	-	-	-	-	-	-
LEU193	0.94	16.16	0.77	0.62	19.77	0.89	m3	0.83	-	-	-	-	-	-	-
LEU194	0.95	16.87	0.82	0.61	20.56	0.95	m1	0.86	-	-	4.18	0.49	2240.09	413.96	LM63
THR195	0.98	14.31	0.80	0.63	17.47	0.89	m5	0.75	0.80	0.95	-	-	-	-	-
GLY196	1.12	13.20	0.81	0.77	14.96	0.89	m5	0.63	0.81	0.78	-	-	-	-	-

Continued on next page

Table 2 – continued from previous page

	**600 MHz**			**850 MHz**			**Model Free**				**CPMG**				
Residue	R1	R2	NOE	R1	R2	NOE	Model	S^2	S^2_f	S^2_s	R^2_{eff}	$\delta R^2_{\mathrm{eff}}$	k_{ex}	δk_{ex}	Model
GLU197	0.87	14.26	0.72	0.55	17.15	0.76	m5	0.74	0.76	0.97	-	-	-	-	-
LEU198	0.82	12.19	0.51	0.55	15.86	0.55	m5	0.65	0.73	0.89	-	-	-	-	-
LEU199	0.92	16.56	0.68	0.59	19.72	0.82	m5	0.81	0.85	0.95	-	-	-	-	-
THR200	0.87	16.47	0.80	0.54	18.45	0.92	m1	0.80	-	-	-	-	-	-	-
LEU201	1.02	15.31	0.85	0.64	18.71	0.88	m1	0.86	-	-	-	-	-	-	-
ALA202	1.02	16.86	0.85	0.65	19.75	0.90	m1	0.86	-	-	-	-	-	-	-
SER203	1.00	17.61	0.88	0.61	19.13	0.93	m1	0.86	-	-	-	-	-	-	-
ARG204	1.00	16.96	0.89	0.69	21.91	0.90	m3	0.87	-	-	-	-	-	-	-
GLN205	1.00	16.60	0.81	0.66	20.89	0.92	m3	0.88	-	-	-	-	-	-	-
GLN206	0.99	16.93	0.82	0.64	19.74	0.91	-	-	-	-	-	-	-	-	-
LEU207	1.08	16.90	0.80	0.67	19.59	0.93	m5	0.84	0.88	0.95	-	-	-	-	-
ILE208	1.03	18.19	0.86	0.67	21.69	0.96	m3	0.89	-	-	-	-	-	-	-
ASP209	1.01	17.77	0.85	0.68	21.03	0.94	m1	0.92	-	-	-	-	-	-	-
TRP210	1.02	16.62	0.81	0.65	20.44	0.91	m3	0.88	-	-	-	-	-	-	-
MET211	1.06	19.77	0.81	0.67	22.51	0.93	m1	1.00	-	-	-	-	-	-	-
GLU212	1.04	46.72	0.83	0.69	-	0.95	m1	0.94	-	-	16.26	8.81	-	-	None
ALA213	0.89	18.49	0.71	0.62	33.35	0.85	m4	0.77	-	-	15.14	1.77	2460.57	320.28	LM63
ASP214	1.14	27.46	0.79	0.60	57.76	1.28	m3	1.00	-	-	15.14	7.72	-	-	None
LYS215d	1.28	6.92	1.52	0.26	-	0.07	-	-	-	-	30.71	6.14	-	-	None
VAL216d	1.61	60.82	0.86	0.68	-	0.63	-	-	-	-	-	-	-	-	-
ALA217	1.03	18.14	0.75	0.60	26.73	1.01	m3	0.85	-	-	11.83	3.00	2670.10	664.52	LM63
GLY218	0.93	38.31	0.78	0.67	57.84	1.07	m3	0.96	-	-	17.61	11.10	2322.80	3648.34	LM63
PRO219	-	-	-	-	-	-	-	-	-	-	-	-	-	-	-
LEU220d	0.74	15.27	0.74	0.81	15.77	0.91	-	-	-	-	17.67	9.54	-	-	None
LEU221d	0.90	56.94	0.87	0.54	4.33	1.18	-	-	-	-	5.25	1.05	-	-	None
ARG222d	0.73	17.77	0.70	0.59	39.90	0.75	-	-	-	-	-	-	-	-	-
SER223d	1.13	17.67	0.48	0.64	20.48	0.93	-	-	-	-	-	-	-	-	-
ALA224	0.92	15.40	0.75	0.65	19.50	0.83	m5	0.81	0.82	0.98	-	-	-	-	-
LEU225	0.90	17.50	0.78	0.60	20.69	0.89	m3	0.83	-	-	-	-	-	-	-
PRO226	-	-	-	-	-	-	-	-	-	-	-	-	-	-	-
ALA227b	0.96	15.52	0.83	0.61	19.43	0.89	-	-	-	-	-	-	-	-	-
GLY228	0.81	14.38	0.78	0.54	17.18	0.87	m1	0.73	-	-	-	-	-	-	-
TRP229	0.93	16.76	0.79	0.65	19.37	0.97	m1	0.92	-	-	-	-	-	-	-
PHE230	0.92	18.14	0.84	0.61	23.60	0.93	m3	0.82	-	-	-	-	-	-	-
ILE231	0.99	17.35	0.85	0.63	23.92	0.94	m3	0.87	-	-	4.23	0.53	2361.15	376.49	LM63

Continued on next page

Table 2 – continued from previous page

	**600 MHz**			**850 MHz**			**Model Free**				**CPMG**				
Residue	R1	R2	NOE	R1	R2	NOE	Model	S^2	S^2_f	S^2_s	$R^2{}_{\text{eff}}$	$\delta R^2{}_{\text{eff}}$	k_{ex}	δk_{ex}	Model
ALA232	0.90	18.41	0.88	0.56	27.36	0.83	m3	0.81	-	-	8.18	1.53	1969.83	323.05	LM63
ASP233	0.92	17.96	0.87	0.61	25.78	0.93	m3	0.80	-	-	6.57	0.65	2217.91	250.46	LM63
LYS234d	0.43	-	1.05	0.51	-	-	-	-	-	-	40.45	8.09	-	-	None
SER235	1.31	59.28	0.89	0.72	62.56	0.88	m4	0.87	-	-	10.88	2.18	-	-	None
GLY236d	0.94	21.31	0.78	0.57	22.67	0.96	-	-	-	-	37.03	7.41	-	-	None
ALA237a	-	-	-	-	-	-	-	-	-	-	-	-	-	-	-
SER238a	-	-	-	-	-	-	-	-	-	-	-	-	-	-	-
GLU240a	-	-	-	-	-	-	-	-	-	-	-	-	-	-	-
ARG241a	-	-	-	-	-	-	-	-	-	-	-	-	-	-	-
GLY242a	-	-	-	-	-	-	-	-	-	-	-	-	-	-	-
SER243a	-	-	-	-	-	-	-	-	-	-	-	-	-	-	-
ARG244a	-	-	-	-	-	-	-	-	-	-	-	-	-	-	-
GLY245a	-	-	-	-	-	-	-	-	-	-	-	-	-	-	-
ILE246	0.84	50.85	0.93	0.59	88.54	0.99	m3	0.81	-	-	21.58	9.91	-	-	None
ILE247a	-	-	-	-	-	-	-	-	-	-	-	-	-	-	-
ALA248	0.97	16.33	0.88	0.62	20.25	0.93	m1	0.87	-	-	-	-	-	-	-
ALA249	0.94	16.62	0.81	0.56	19.54	0.93	m1	0.81	-	-	-	-	-	-	-
LEU250	1.00	17.19	0.80	0.64	18.94	0.89	m1	0.90	-	-	-	-	-	-	-
GLY251	0.94	16.97	0.83	0.66	20.80	0.95	m3	0.84	-	-	-	-	-	-	-
PRO252	-	-	-	-	-	-	-	-	-	-	-	-	-	-	-
ASP254	0.94	19.62	0.79	0.62	26.26	0.95	m3	0.82	-	-	4.19	0.45	-	-	None
GLY255	1.05	22.80	0.82	0.69	30.64	0.86	m3	0.94	-	-	-	-	-	-	-
LYS256	0.94	17.72	0.80	0.62	21.59	0.91	m3	0.87	-	-	-	-	-	-	-
PRO257	-	-	-	-	-	-	-	-	-	-	-	-	-	-	-
SER258c	1.07	19.03	0.78	0.62	22.58	0.86	-	-	-	-	-	-	-	-	-
ARG259	1.00	17.60	0.86	0.69	23.66	0.91	m3	0.84	-	-	4.27	0.58	2360.65	428.17	LM63
ILE260	0.95	16.22	0.79	0.62	20.94	0.98	m3	0.84	-	-	-	-	-	-	-
VAL261	0.90	16.21	0.84	0.59	22.37	0.91	m3	0.78	-	-	4.16	0.45	1982.99	313.62	LM63
VAL262	0.87	16.06	0.79	0.55	20.23	0.88	m3	0.80	-	-	-	-	-	-	-
ILE263	0.88	17.36	0.81	0.53	20.78	0.88	m3	0.80	-	-	-	-	-	-	-
TYR264	0.88	19.91	0.84	0.60	28.31	0.91	m3	0.81	-	-	11.57	1.20	1467.33	162.38	LM63
THR265	0.88	31.14	0.78	0.56	48.40	0.81	m3	0.81	-	-	11.23	4.13	1048.00	133.56	LM63
THR266a	-	-	-	-	-	-	-	-	-	-	-	-	-	-	-
GLY267a	-	-	-	-	-	-	-	-	-	-	-	-	-	-	-
SER268	0.92	23.55	0.79	0.64	33.33	0.77	m4	0.80	-	-	11.98	1.73	2682.76	592.59	LM63

Continued on next page

Table 2 – continued from previous page

	**600 MHz**			**850 MHz**			**Model Free**				**CPMG**				
Residue	R1	R2	NOE	R1	R2	NOE	Model	S^2	S^2_f	S^2_s	$R^2{}_{\mathrm{eff}}$	$\delta R^2{}_{\mathrm{eff}}$	k_{ex}	δk_{ex}	Model
GLN269	0.90	18.43	0.95	0.62	24.47	0.98	m3	0.84	-	-	11.23	0.99	744.56	309.67	CR72
ALA270	1.01	13.33	0.83	0.65	22.49	0.87	m1	0.86	-	-	-	-	-	-	-
THR271	0.93	18.56	0.81	0.58	22.85	0.84	m4	0.84	-	-	-	-	-	-	-
MET272	0.93	21.75	0.97	0.70	27.85	0.95	m3	0.89	-	-	13.09	6.99	1458.41	1494.11	LM63
ASP273	0.94	17.41	0.85	0.60	22.16	0.95	m3	0.82	-	-	-	-	-	-	-
GLU274	0.97	18.74	0.73	0.64	22.48	0.86	m4	0.87	-	-	-	-	-	-	-
ARG275	1.05	20.12	0.80	0.67	26.20	0.89	m3	0.94	-	-	5.50	0.91	555.41	201.22	LM63
ASN276	0.96	18.23	0.76	0.59	24.77	0.94	m4	0.84	-	-	6.26	0.84	952.37	196.61	LM63
ARG277	1.00	21.78	0.77	0.66	31.73	0.95	m4	0.87	-	-	8.32	0.85	2524.77	245.24	LM63
GLN278	0.99	14.62	0.48	0.63	21.92	0.85	m5	0.73	0.83	0.88	6.28	0.40	187.88	87.14	LM63
ILE279	0.95	19.26	0.87	0.60	25.43	0.95	m3	0.84	-	-	6.41	2.14	1341.77	1276.51	LM63
ALA280c	1.11	17.24	0.87	0.72	23.60	0.88	-	-	-	-	4.17	0.86	136.63	215.12	CR72
GLU281	1.28	20.00	0.89	0.66	22.02	0.91	m1	0.98	-	-	-	-	-	-	-
ILE282	1.02	19.43	0.86	0.61	23.37	0.91	m3	0.91	-	-	4.80	1.01	2578.45	847.10	LM63
GLY283	0.97	16.19	0.86	0.62	20.64	0.89	m1	0.87	-	-	-	-	-	-	-
ALA284	1.02	17.42	0.83	0.68	23.59	0.91	m3	0.90	-	-	5.15	0.44	1743.18	327.21	CR72 full
SER285	0.99	17.09	0.83	0.58	19.73	0.91	m1	0.85	-	-	-	-	-	-	-
LEU286c	0.92	15.87	0.78	0.61	21.29	0.92	-	-	-	-	-	-	-	-	-
ILE287	1.05	23.84	0.96	0.63	31.17	0.98	m3	0.91	-	-	10.00	2.06	798.13	564.86	CR72
LYS288	1.00	16.93	0.84	0.66	20.03	0.93	m1	0.90	-	-	-	-	-	-	-
HIS289	0.87	17.32	0.87	0.54	20.93	0.94	m3	0.81	-	-	-	-	-	-	-
TRP290	0.95	13.80	0.78	0.63	15.89	0.92	m5	0.70	0.74	0.94	-	-	-	-	-

TABLE 3: HDX-MS Summary Table

Data Set	TEM-1	G238S	E104K/G238S	A42G/E104K/G238S
HDX reaction details	25 mM MES, pD_{read} = 7.1, 15 °C	25 mM MES, pDread = 7.1, 15 °C	25 mM MES, pDread = 7.1, 15 °C	25 mM MES, pDread = 7.1, 15 °C
HDX time course (min)	0.5, 1.67, 16.67, 50, 150, 250	0.5, 1.67, 16.67, 50, 150, 250	0.5, 1.67, 16.67, 50, 150, 250	0.5, 1.67, 16.67, 50, 150, 250
HDX control samples	Undeuterated control (WT protein)	Undeuterated control (G238S protein)	Undeuterated control (E104K/G238S protein)	Undeuterated control (A42G/E104K/G238S protein)
Back-exchange (mean / IQR)	n/a		n/a	
# of Peptides	74 (high conf.)	74 (high-conf.)	74(high-conf.)	74(high-conf.)
Sequence coverage	96%	96%	96%	96%
Average peptide length / Redundancy	7.3 / 3.7	7.3 / 3.7	7.3 / 3.7	7.3 / 3.7
Replicates (biological or technical)	3 (technical)	1 (technical)	1 (technical)	1 (technical)
Repeatability	0.15 (average standard deviation)	n/a	n/a	n/a
Significant differences in HDX (delta HDX > X D)	0.25 D		0.25 D	
